# Online Ergonomic Evaluation in Realistic Manual Material Handling Task: Proof of Concept

**DOI:** 10.3390/bioengineering11010014

**Published:** 2023-12-23

**Authors:** Sergio Leggieri, Vasco Fanti, Darwin G. Caldwell, Christian Di Natali

**Affiliations:** 1 Department of Advanced Robotics, Istituto Italiano di Tecnologia, 16163 Genova, Italy; vasco.fanti@iit.it (V.F.); darwin.caldwell@iit.it (D.G.C.); christian.dinatali@iit.it (C.D.N.); 2 Department of Informatics, Bioengineering, Robotics and Systems Engineering (DIBRIS), Università degli Studi di Genova (UniGe), 16145 Genova, Italy

**Keywords:** ergonomic risk assessment, event detection, biomechanical analysis, NIOSH, work-related musculoskeletal disorders, wearable technology

## Abstract

Work-related musculoskeletal disorders are globally one of the leading causes of work-related injuries. They significantly impact worker health and business costs. Work task ergonomic risk indices have been developed that use observational assessments to identify potential injuries, and allow safety managers to promptly intervene to mitigate the risks. However, these assessments are very subjective and difficult to perform in real time. This work provides a technique that can digitalize this process by developing an online algorithm to calculate the NIOSH index and provide additional data for ergonomic risk assessment. The method is based on the use of inertial sensors, which are easily found commercially and can be integrated into the industrial environment without any other sensing technology. This preliminary study demonstrates the effectiveness of the first version of the Online Lifting Index (On-LI) algorithm on a common industrial logistic task. The effectiveness is compared to the standard ergonomic assessment method. The results report an average error of 3.6% compared to the NIOSH parameters used to calculate the ergonomic risk and a relative error of the Lifting Index of 2.8% when compared to observational methods.

## 1. Introduction

Worker quality of life is crucial across all industrial fields; however, work-related musculoskeletal disorders (WMSDs) are having a worldwide impact on worker health [1,2]. The European Agency for Safety and Health at Work survey found that, in Europe, 60% of work-related health problems affect the musculoskeletal system, and the back is the most affected area [3]. Reducing or preventing these disorders would significantly impact companies and society from both a well-being and an economic perspective, since reducing WMSDs will result in a decrease in absenteeism, and a saving of money and manpower [4,5,6,7]. National surveys in the U.K. [8] and Germany [9] reported GBP 5.7 billion of production losses and more than EUR 30 billion of gross losses due to musculoskeletal disorders in the upper body. To prevent/reduce these costs companies rely on ergonomists and qualified personnel that, through field inspections, assess operating environments and movements performed by workers. Based on observational measures, they can calculate ergonomic risk indices. These risk indices differ according to the tasks performed and consider both the kind of activity executed (static, dynamic, repetitive, etc.) and the body segment involved. For example, to evaluate the risk of WMSDs, and thus the injury-free lifting capabilities during manual material handling (MMH) activities, the NIOSH lifting equations [10,11,12] are used, while the TACOS [13] is the most appropriate to assess static and dynamic postural aspects. For workers performing repetitive movements and exertions of the upper limbs, relevant assessment methods are the OCRA [14], the Strain Index [15], and the RULA. The last three methods include subjective data to quantify the effort required to accomplish the task, and they have poor repeatability, making them unsuitable for objective analysis [16]. As employers have legal (and ethical) obligations to avoid/limit worker exposure to manual handling of loads, evaluation of the ergonomic risk of specific work tasks is vital in assisting the whole ergonomic analysis and redesign of the work environment. In fact, to reduce the chance of injury, work tasks should be designed to limit exposure to ergonomic risk factors. Engineering controls are the most desirable, where possible [17].

This paper focuses on MMH, and the associated work tasks regulated by ISO 11228-1, [18], namely: lifting, carrying, and lowering. The MMH ergonomic assessment is carried out in an observational manner using the NIOSH method to calculate the risk indices. A digital ergonomic assessment is fundamental to deliver a more consistent risk evaluation [19,20,21,22]. From the literature, several works [22,23,24] aim to evaluate the ergonomic risk associated with the upper limbs for human–robot interaction purposes. Some studies provide an online risk evaluation, but they use equipment that is not well suited for real adoption in industrial scenarios, such as force sensors to calculate ground reaction forces [20], high-resolution cameras for motion capture [19], or box sensing to track the movement of boxes [25]. More recently, Lorenzini et al. [26] have suggested evaluating ergonomic risk factors using robotics-inspired indices. Other works, based on ergonomic assessment worksheets (EAWSs), make use of data from inertial suits and sensorized gloves to train recognition models based on hidden Markov models to provide online risk evaluation, as in [27,28,29]. However, these are still not correlated to worldwide accepted standard ergonomic assessment methods as cited in ISO 11228-1:2022 [30].

**Figure 1:**
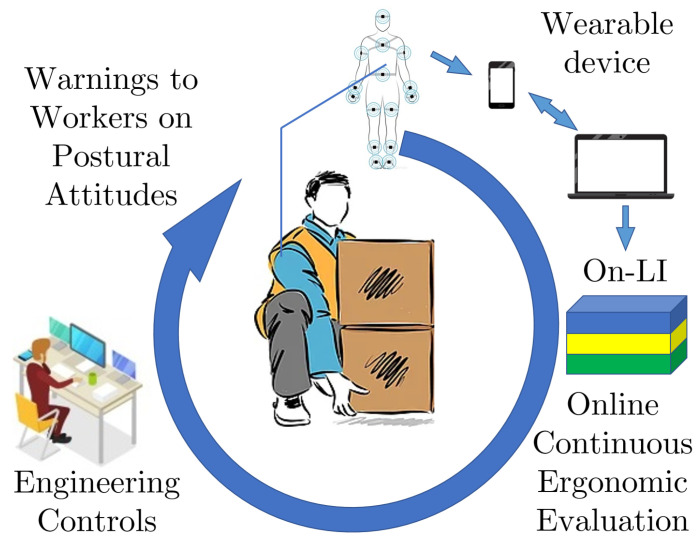
Human in the loop. This work aims at improving working conditions by providing online ergonomic evaluations of the NIOSH Lifting Index.

This study uses a set of sensors that can be included in a wearable device for online ergonomic assessment in MMH. Here, data from only inertial sensors are used together with kinematic-based algorithms to assess a worker’s postures during logistics activities. A proof of concept is proposed to provide online ergonomic assessment of the NIOSH Lifting Index. The algorithm identifies lifting and release instants of each activity and monitors any incongruous or dangerous postural behavior and warns the worker. The introduction of online ergonomic teaching features contributes to a safer industrial work environment, as introduced in Figure 1. Indeed, digitalization of the ergonomic risk assessment enables a standardized evaluation procedure based on stable data, allowing comparisons between workers or within multiple activities performed by the same worker. Moreover, a digital ergonomic evaluation, if recorded over a long period, allows the ergonomist to support the assessment with a statistical analysis. Several essential services would be enabled when considering extending the ergonomic risk assessment from a single measurement event to a continuum measurement supported by a non-invasive wearable device. Online ergonomic assessment will enable monitoring of the risk evolution over time and worker training during work shifts by eliminating risky postural attitudes; see Figure 1. This preliminary study has the goal of improving the quality of working conditions for workers according to the civil construction and railway sector requirements mentioned in [31]. In this paper, Section 2 defines the general framework and details the innovative architecture for the assessment of the NIOSH lifting index. Section 3 describes the experimental protocol used to validate our ergonomic evaluation method. The results are presented in Section 4 and discussed in Section 5. Finally, Section 6 offers some concluding remarks and directions for future work.

## 2. Materials and Methods

### 2.1. On-LI Architecture

**Figure 2:**
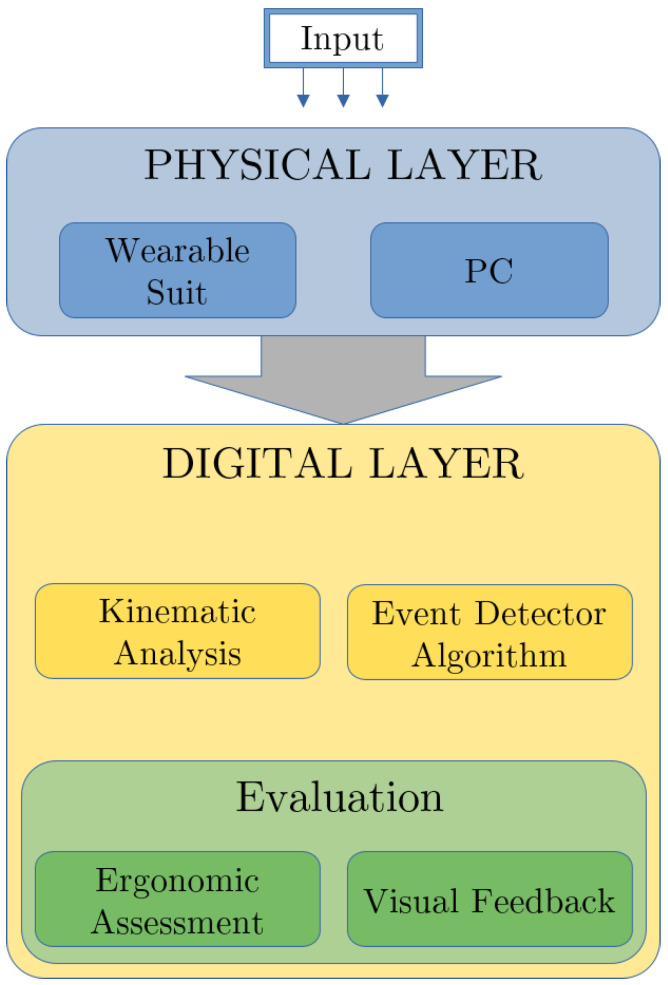
The On-LI algorithm uses NIOSH evaluation criteria to provide an online assessment of the ergonomic risks associated with the MMH task.

The Online Lifting Index (On-LI) architecture proposed in this work consists of two interconnected layers, as shown in Figure 2. The On-LI algorithm exchanges several inputs internally and data of different natures, such as inertial, kinematic, geometrical, and temporal information, to produce an accurate assessment of the ergonomic risks associated with MMH activities. In this scheme, the first layer can be considered as the physical level. It is formed from the wearable IMU sensors and the processing unit. These sensors acquire the subject-specific inertial measurements and the processing unit collects and fuses these data into the digital layer. The digital layer represents the central core of this work, and it is responsible for the data processing and the evaluation level. The main blocks of the digital layer are: the kinematic analysis, the event detector algorithm (EDA), and the evaluation blocks. The kinematic analysis and the EDA are strongly interconnected and fetch all data for the evaluation level. Finally, the evaluation level includes the ergonomic assessment and the visual feedback of the On-LI system. The On-LI has been developed to work online, producing assessments of the NIOSH Lifting Index and visual feedbacks of the most critical postures adopted by subjects. At the moment, the On-LI’s main limitation is the wearable sensors currently used, which do not allow online data sharing. The future goal would be to enable real-time communication with the wearable sensors to enhance the On-LI by ensuring real online and continuous assessments of the NIOSH Lifting Index and relevant warnings for workers. This work focuses on verifying the effectiveness of our online ergonomic evaluation method through simulations that emulate real-time data sharing, and comparing the results against the traditional observational method.

### 2.2. Kinematic Analysis

**Figure 3:**
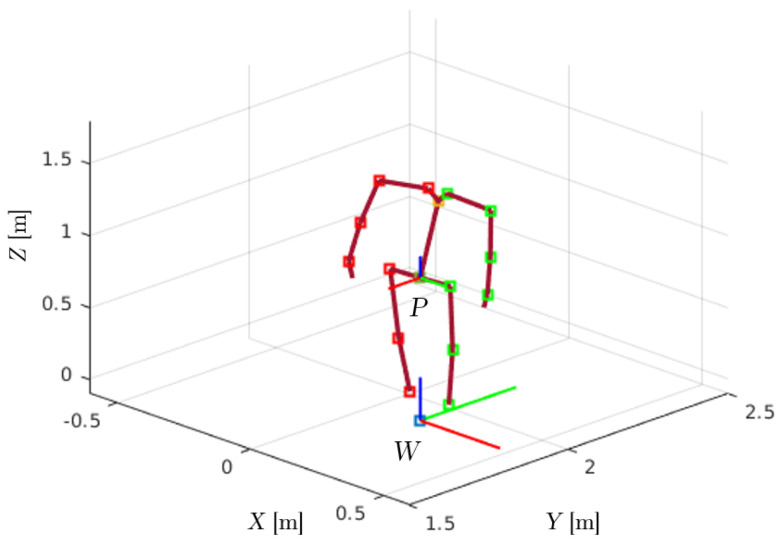
The biomechanical model of the On−LI system. Such a model reconstructs the subject posture. The dark red lines represent the body segments, the red and green squares represent the body joints of the right and left sides, respectively. The inertial reference is the world frame W, whose *x*, *y* and *z* axes are represented by the red, green and blue lines, respectively; on the pelvis there is the local frame *P*, whose *x*, *y* and *z* axes are represented by the red, green and blue lines, respectively.

The data gathered and shared by the physical level of the On-LI architecture is fed to the kinematic analysis block. Here, the kinematic variables are processed. The position $p_{i}^{W}\in R^{3}$ and the orientation matrix $R_{i}^{W}\in SO\left(3\right)$ of each *i*-th link are used to reconstruct the biomechanical model of each subject. The model consists of i=14 links connected by eleven spherical joints and a “virtual” joint with six degrees of freedom (DoFs). During the functional calibration of the wearable suit, the world frame *W* is established with the *x*-axis pointing forward, the *y*-axis pointing to the left, and the *z*-axis pointing upward, as shown in Figure 3. A local frame is attached to the middle of each link in the model, starting from the pelvis frame *P*, which is placed in the midpoint of the right and left *antero-superior iliac spine*. The “virtual” joint relates the instantaneous position and orientation of *P* with respect to frame *W*. It consists of three prismatic joints for the linear displacement and a spherical joint for the orientation. The biomechanical model can be considered a kinematic chain with branches. From frame *P*, three branches spread toward the trunk and toward the right and left hips. Two additional branches originate from trunk frame *T* and connect to the right and left shoulders. Given the pelvis position vector $p_{P}^{W}\left(t\right)$, the biomechanical model is generated iteratively as follows:

(1) $$p_{Rhip}^{W}\left(t\right)=p_{P}^{W}\left(t\right)+R_{P}^{W}\left(t\right)l_{hip}^{P}$$

where the vector $p_{P}^{W}\left(t\right)$ and the matrix $R_{P}^{W}\left(t\right)$ represent the absolute position and orientation of frame *P* with respect to frame *W*, respectively. The variable $p_{Rhip}^{W}$ represents the right hip position vector expressed in frame *W* coordinates. The vector $l_{hip}^{P}$ represents the length from the pelvis to the hips, expressed in *P* frame coordinates. In this way, all segments of the biomechanical model are expressed in frame *W* coordinates. However, the subject’s overall orientation may vary widely while performing MMH activities, especially about the *z*-axis of frame *W*. This makes the kinematic analysis in *W* coordinates cumbersome. Therefore, an additional “virtual” frame *B* is considered and each hand position and velocity vector is projected onto it. Frame *B* can be thought of as a frame attached to the grasped object. The orientation of Frame *B* is estimated starting from the orientation on each hand. The transformation matrix ${\hat{R}}_{B}^{W}\left(t\right)$ results in

(2) $${\hat{R}}_{B}^{W}\left(t\right)=\left[\begin{array}{ccc} cos\left({\bar{q}}_{H}\right) & -sin\left({\bar{q}}_{H}\right) & 0\\ sin\left({\bar{q}}_{H}\right) & cos\left({\bar{q}}_{H}\right) & 0\\ 0 & 0 & 1 \end{array}\right]$$

which corresponds to a rotation of ${\bar{q}}_{h}$ radians about the *z*-axis of frame *W*. The variable ${\bar{q}}_{H}\left(t\right)$ corresponds to the average of the right, $q_{RHa_{z}}\left(t\right)$, and left, $q_{LHa_{z}}\left(t\right)$, angles of the hands about the *z*-axis of frame *W*. The position and velocity vectors $p_{RHa}^{W}$, $v_{RHa}^{W}$, $p_{LHa}^{W}$, and $v_{LHa}^{W}$ of the right and left hands, respectively, are projected in frame *B* coordinates as follows:

(3) $$\begin{array}{ccc} p_{RHa}^{B}\left(t\right) & = & {\hat{R}}_{B}^{W}{\left(t\right)}^{T}p_{RHa}^{W}\left(t\right)\\ v_{RHa}^{B}\left(t\right) & = & {\hat{R}}_{B}^{W}{\left(t\right)}^{T}v_{RHa}^{W}\left(t\right) \end{array}$$

where ${\hat{R}}_{B}^{W}{\left(t\right)}^{T}$ is the inverse of matrix ${\hat{R}}_{B}^{W}\left(t\right)$. This work targets MMH activities, and therefore, the relative distance, as well as the average and the relative velocities of the hands, represent key variables for identifying the grasping, lifting, and release phases. From the difference $\Delta p_{H}^{B}\left(t\right)=p_{RHa}^{B}\left(t\right)-p_{LHa}^{B}\left(t\right)$, it is possible to estimate the instantaneous relative distance $d\left(t\right)$ between the hands as follows:

(4) $$d\left(t\right)=\sqrt[{2}]{\Delta p_{H}^{B}{\left(t\right)}^{T}\Delta p_{H}^{B}\left(t\right)}.$$

This variable is relevant in identifying the load grasping. The average velocity vector ${\bar{v}}_{H}^{B}\left(t\right)$ is given by

(5) $${\bar{v}}_{H}^{B}\left(t\right)=\frac{v_{RHa}^{B}\left(t\right)+v_{LHa}^{B}\left(t\right)}{2}.$$

In particular, the *z* component of ${\bar{v}}_{H}^{B}\left(t\right)$ assumes relevance in identifying the load lifting instant. Finally, the hand’s relative velocity vector $\Delta v_{H}^{B}\left(t\right)$ is computed as follows:

(6) $$\Delta v_{H}^{B}\left(t\right)=v_{LHa}^{B}\left(t\right)-v_{RHa}^{B}\left(t\right).$$

Specifically, the *y* component of $\Delta v_{H}^{B}\left(t\right)$ can be thought of as the normal velocity with respect to the load. Positive values of $\Delta v_{H_{y}}^{B}\left(t\right)$ are associated with the load release. The kinematic analysis block routes these three variables to the event detector algorithm. In addition, this block connects to the ergonomic assessment and provides it with the pelvis, right/left hip, and knee flexion/extension angles.

### 2.3. Event Detector Algorithm

**Figure 4:**
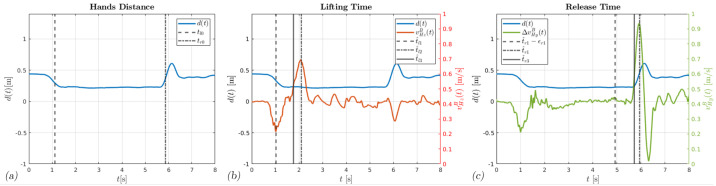
The EDA estimations. In (**a**), the distance $d\left(t\right)$ between hands is shown in blue, the lifting time estimate ${\hat{t}}_{l0}$ is the dashed line, and the release time estimate ${\hat{t}}_{r0}$ is the dash−dot line. In (**b**), the *z* component of the average velocity vector ${\bar{v}}_{H}^{B}\left(t\right)$, the estimate ${\hat{t}}_{r1}-\epsilon_{r1}$ is the dashed line, the estimate ${\hat{t}}_{r1}$ is the dash−dot line, and the estimate ${\hat{t}}_{r3}$ is the solid line. In (**c**), the *y* component of the hands relative velocity vector $\Delta v_{H}^{B}\left(t\right)$, the estimate ${\hat{t}}_{r1}-\epsilon_{r1}$ is the dashed line, the estimate ${\hat{t}}_{r1}$ is the dash-dot line, and the estimate ${\hat{t}}_{r3}$ is the solid line.

As the name suggests, the event detector algorithm is responsible for the estimation of the instants when the lift and release occur; $t_{l}$ and $t_{r}$ are the times of these events, respectively. Timing is very critical for identifying correctly the instantaneous posture of the subject to perform accurate ergonomic evaluations of MMH activities with only inertial sensors. For this, the EDA fuses the information of the hand’s distance $d\left(t\right)$, the average velocity ${\bar{v}}_{H}^{B}\left(t\right)$, and the relative velocity $\Delta v_{H}^{B}\left(t\right)$ to progressively refine the estimates of $t_{l}$ and $t_{r}$. The algorithm works as follows. For identifying the lifting instant $t_{l}$, the algorithm compares the distance $d\left(t\right)$ with a given grasp threshold ${\tilde{d}}_{g}$, as shown in Figure 4a. If the grasp occurs along the shortest edge of the load, the algorithm produces the first approximation ${\hat{t}}_{l0}$. If the load is below the level of the subject hands, the *z* component of the average velocity ${\bar{v}}_{H}^{B}\left(t\right)$ presents a negative peak when the subject bends, followed by a positive peak when the subject lifts the load, as shown in Figure 4b. The EDA scans the *z* component of the average velocity ${\bar{v}}_{H}^{B}\left(t\right)$ in the interval $t\in \left[{\hat{t}}_{l0}-\epsilon_{l};{\hat{t}}_{l0}+\epsilon_{l}\right]$ looking for those two peaks. The variable $\epsilon_{l}>0$ defines the time interval being investigated. The algorithm identifies the two instants ${\hat{t}}_{l1}$ and ${\hat{t}}_{l2}$ that correspond to the minimum and maximum peaks, respectively. Finally, the EDA scans ${\bar{v}}_{Hz}^{B}\left(t\right)$ again in the new interval $t\in \left[{\hat{t}}_{l1};{\hat{t}}_{l2}\right]$, and compares it to a given threshold ${\tilde{v}}_{Hz}^{B}>0$. If the condition is met, the algorithm determines the last estimate ${\hat{t}}_{l3}$. Comparing Figure 4a,b, the difference between the first estimate ${\hat{t}}_{l0}$ and the final estimate ${\hat{t}}_{l3}$ is clear. In this case, the real lifting event occurs almost 0.8 seconds later than ${\hat{t}}_{l0}$.

The EDA performs a similar procedure to identify the release instant $t_{r}$. It compares the distance $d\left(t\right)$ with a given release threshold ${\tilde{d}}_{r}$ and detects the instant ${\hat{t}}_{r0}$. In this case, the algorithm scans the *y* component of the relative velocity $\Delta v_{H}^{B}\left(t\right)$ in the interval $t\in \left[{\hat{t}}_{r0}-\epsilon_{r},{\hat{t}}_{r0}+\epsilon_{r}\right]$. Here, the variable $\epsilon_{r}>0$ defines the interval width. As shown in Figure 4c, the velocity component $\Delta v_{Hy}^{B}\left(t\right)$, which is normal with respect to the grasped object, presents a positive peak when the subject releases the load and detaches the hands. Usually, this peak is followed by another one when the subject returns the arms to the resting position. The EDA identifies the instants when the positive, ${\hat{t}}_{r1}$, and the negative, ${\hat{t}}_{r2}$, peaks occur. Then, the algorithm discards the instant ${\hat{t}}_{r2}$ and refines the search interval into $\left[{\hat{t}}_{r1}-\epsilon_{r1};{\hat{t}}_{r1}\right]$. The variable $\epsilon_{r1}>0$ specifies the time span investigated. Lastly, the algorithm verifies when the *y* component of $\Delta v_{H}^{B}\left(t\right)$ crosses a given threshold $\Delta {\tilde{v}}_{H}^{B}>0$ in this interval. When the condition is met, the algorithm produces the last estimate, ${\hat{t}}_{r3}$. Again, comparing Figure 4a,c, the difference between ${\hat{t}}_{r0}$ and ${\hat{t}}_{r3}$ amounts to almost 0.2 s.

### 2.4. Ergonomic Assessment: Digital Algorithm

The NIOSH Lifting Index provides a risk factor associated with a given MMH task. Currently, all NIOSH parameters are measured or evaluated manually, introducing possible human errors or over-simplifications in the process. Additionally, the assessment is only conducted on a single or a small number of workers. The ergonomic assessment block within the new framework relies on previous steps of the On-LI algorithm to generate precise assessments of the Lifting Index. For all the identified lift and release instants ${\hat{t}}_{l3}$ and ${\hat{t}}_{r3}$, the NIOSH parameters are evaluated and combined with the corresponding kinematic data, resulting in a single value known as the recommended weight limit (RWL). This is calculated as follows:

(7) RWL=wc×hd×vh×vd×af×f×gf.

The variable wc is the maximum weight allowed. The parameters hd and vh are the horizontal and vertical distances between the load position when lifted and the center of the worker’s ankles. vd represents the vertical displacement of the hands between the beginning and end of the task. The asymmetry factor af considers the load angular displacement with respect to the worker’s sagittal plane, i.e., the upper body twist. The frequency f is computed as the number of objects lifted per minute. Finally, the grip factor gf encodes the type of grasp: good (G), sufficient (S), or poor (P). The Lifting Index (LI) provides a standardized risk index for a given task, and it is computed as

(8) $$\mathrm{LI}=\frac{\mathrm{WL}}{\mathrm{RWL}}$$

where the variable WL represents the weight of the load lifted. As listed in Table 1, LI values below 1.5 indicate a low injury risk, while higher values denote moderate to very high risks [10,11,12].

### 2.5. Visual Feedback

**Figure 5:**
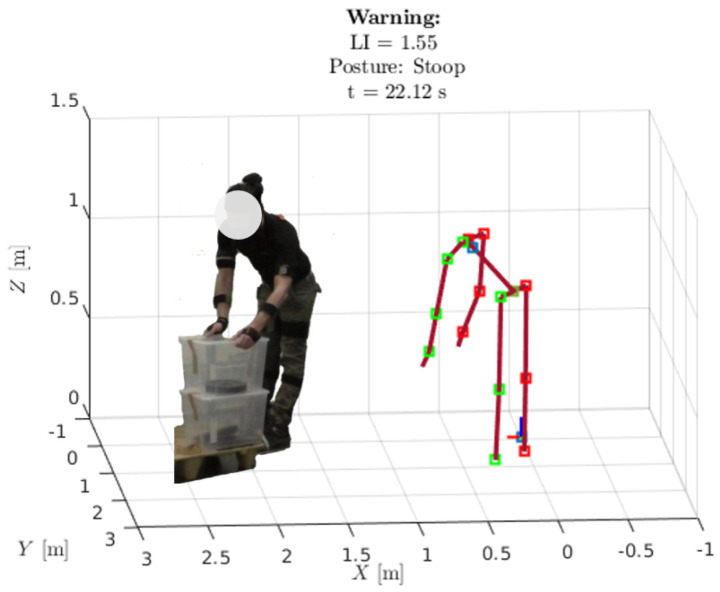
Visual feedback output. The biomechanical model reconstructs the subject’s posture. The On−LI algorithm provides a LI warning and a posture warning.

Referring to Figure 2, the ergonomic assessment algorithm also estimates the subject’s posture while lifting the object. The pelvis, the right/left hip, and the knee flexion angles ($q_{P}$,$q_{RHip}$, $q_{LHip}$, $q_{RK}$, $q_{LK}$) are evaluated. If the subject performs a stoop, e.g., the angles $q_{P}$, $q_{RHip}$, $q_{LHip}$ are above and the angles $q_{RK}$, $q_{LK}$ are below a given threshold ${\tilde{q}}_{lim}$, the ergonomic assessment sends a warning to the visual feedback block. This block visualizes the biomechanical model online and includes any possible warnings generated by the ergonomic assessment block, as shown in Figure 5. Such feedback provides the ergonomist or, in general, the person in charge of safety, with an additional tool to determine the risk of WMSDs. This visual feedback may directly help the subject throughout the activity in adopting safer postures by providing online notification warnings.

## 3. Experimental Assessment

### 3.1. Subjects and Data Collection

For the experiments, we recruited five healthy subjects (age: 29 ± 4 years; height: 184 ± 6 cm; weight: 77 ± 15 kg). To limit discomfort, each subject wore, on top of their clothes, seventeen commercially available wireless inertial motion trackers (MTs) (MTw Xsens technologies, Enschede, The Netherlands). The MTs were used to collect full-body kinematic data and were positioned as reported in the Xsens-Awinda user manual. Precisely, for the lower body segments, one sensor was placed on the pelvis, and six sensors were placed on the mid-thigh, mid-shank, and foot instep, right and left. For the upper body, one sensor was attached on the middle of the sternum, two sensors were placed on the right and left scapulae, one sensor on the back of the head, and six sensors were attached laterally, to the mid-upper arms, to the mid-lower arms, and the back of the hands, right and left. Elastic bands covered each MT to reduce relative movements between sensors and corresponding segments, with the exception of the sensors attached to the scapulae. Data sampling was at 60 Hz. Before the session, each subject was asked to perform the functional calibration procedure to assess the sensors-to-body alignment. In this procedure, each subject maintained a standing upright posture, followed by a walk, a turn on the spot, and a walk back to the starting position.

### 3.2. Experimental Protocol

**Figure 6:**
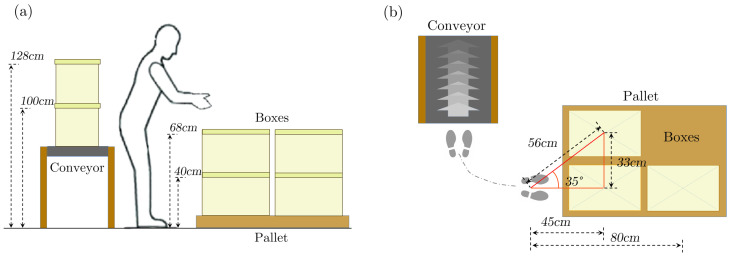
(**a**) Side view and (**b**) top view of the experimental setup reporting the measurements needed to calculate the NIOSH.

The experimental protocol was compliant with the experimental protocol approved by the Ethical Committee of Liguria, Italy, 8 October 2019, protocol number: 001/2019. The experimental session involved simulating a realistic work activity that is common across many industries where logistic tasks are carried out, as reported in our previous works [32,33]. The task involved transferring loads from a pick-up point to a lateral workbench that was two to three steps away. In these experiments, the loads were boxes, the pick-up point was a pallet, and the workbench was a table with a sliding layer to simulate a conveyor. Three stacks of two boxes each were placed precisely on the three corners of the pallet, and each box weighed 10 kg. The first stack was in front and close to the subject, the second, positioned to the left, was placed diagonally with respect to the worker’s sagittal plane, and the third was placed directly behind the first stack but at the far side of the pallet with respect to the subject. According to the order of execution, the boxes were identified by the terms: FH and FL (front stack high and low positions, respectively), LH and LL (lateral stack high and low positions, respectively), and RH and RL (rear stack high and low positions, respectively). The conveyor was on the left relative to the subject’s starting position. The geometry of the experimental environment are shown in Figure 6. The task was to relocate each stack of boxes onto the conveyor. The high box was moved first, then the low box was placed on top, and then the conveyor slid. The order in which the stacks were moved was as follows: first, the one close by; second, the one placed diagonally; and last, the one at the rear. The subjects could select their preferred lifting technique, such as stoop or squat, but they were asked to grab the boxes from the top, along the shortest edge and with both hands. Additionally, the subject had freedom in deciding where to stand with respect to the front stack, within a square tile (40×40 cm). The speed in executing the task was self-selected. At the end of each experimental session, an investigator carried out a traditional ergonomic assessment by applying the NIOSH table equations with the observational method. A video of the experimental section was shot to support the observational evaluation process with visual feedback.

## 4. Results

To validate the proposed methodology, we manually measured all the parameters needed to calculate the LI by applying the NIOSH equation. The observational results are shown in Table 2. The LI associated with FH and FL is below 1.5, while for the other boxes, the LI values range between 1.5 and 2. The same parameters were calculated by importing the Xsens data, implementing the kinematic analysis, the EDA, the ergonomic assessment and the subject-specific kinematic model in MATLAB (R2021b). The corresponding parameters and the ergonomic risk indices are reported in Table 3, which reports the average and the standard deviation of the parameters collected over all workers task by task. With both methods, the LI related to the FH and the FL are below 1.5, and therefore, the ergonomic risk is *low*. The LI related to the LH differs between the observational and the digital method, the former reports a *low* ergonomic risk, while the latter indicates a *moderate* ergonomic risk. Both methods agree in evaluating the risks associated with the remaining boxes as *moderate*.

Table 4 summarizes the absolute errors (Abs.Err.) and the relative errors (Rel.Err.). The Abs. Err. is computed by calculating the difference over each entry of Table 2 and Table 3. The Rel. Err. is calculated by dividing the Abs. Err. by the corresponding full-scale value. Referring only to the linear parameters measured to calculate the LI, the minimum Abs. Err. is 0 cm, while the maximum is 14 cm. Both of these measurements relate to the horizontal distance and the average Abs. Err. is 3.6 cm. The minimum Rel. Err. is 0%, the maximum 22.2%, and the average is 5.8%. For the angular parameters, i.e., the twist, the minimum Abs. Err. is 2 deg, the maximum is 4 deg, and the average is 2.5 deg. The minimum Rel. Err. is 1.4%, the maximum 2.9%, and the average is 1.7%. Concerning the LI, the minimum Abs. Err. is 0.02, the maximum is 0.15, and the average is 0.08. The minimum Rel. Err. is 0.6%, the maximum is 5%, and the average is 2.8%.

## 5. Discussion

The results reported in the previous section are very promising. Most of the parameters and the Lifting Index present marginal absolute and relative errors, less than 10%, as shown in Table 4.

**Figure 7:**
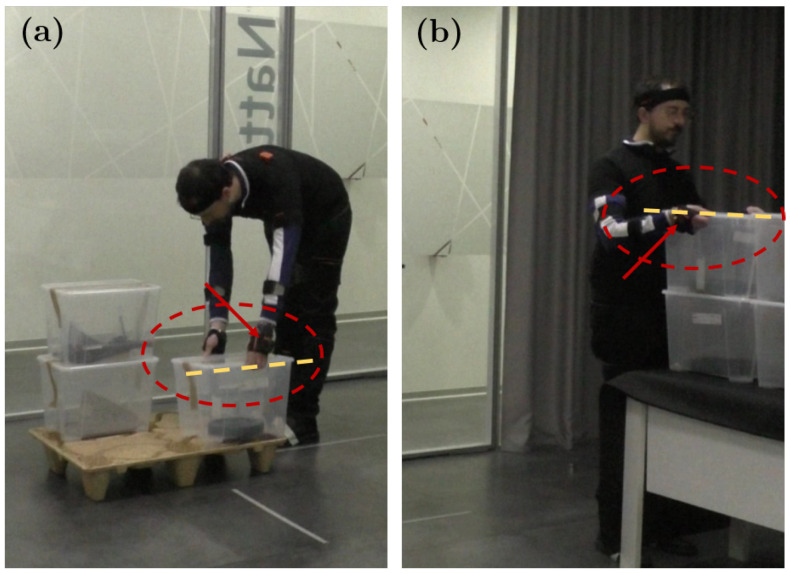
Snapshots from the video footage. In (**a**), the subject picks up the load in position LL. As shown by the red arrow in the circle, the sensors in the gloves are above the box edges, yellow dashed line. In (**b**), the subject releases the load over the previous box. The subjects modified their grasp mid-way through the motion and now the sensors are below the box edges.

**Figure 8:**
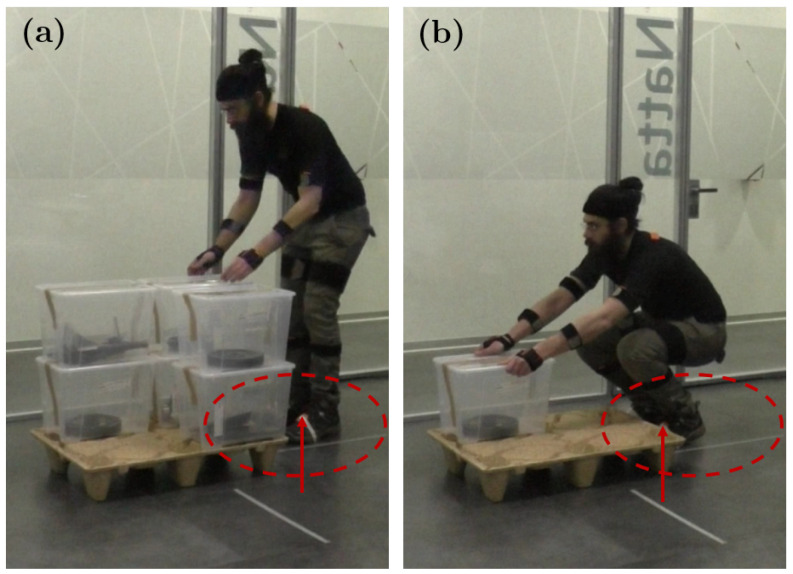
Snapshots from the video footage. In (**a**), the subject picks up the load in position FH while standing far from the pallet. As shown by the red arrow in the circle, the sensors on the feet are visible in the frame. In (**b**), the subject picks up the load in position RL and steps closer to the pallet. In this case, the sensors on the feet are hidden by the pallet.

The most significant relative errors appear on the vertical travel of the lower boxes. Such errors occur because the configurations of the hand and the feet positions change during these activities. Comparing the biomechanical model reconstructions with the corresponding footage, it is easy to notice that the subjects tend to lift the boxes as soon as the fingertips come into contact with the box, then, they adjust the grasp mid-air and, finally, they modify again the grasp configuration to release the load on top of the box previously positioned on the conveyor. These aspects represent a current limitation of the On-LI architecture. In fact, the closest inertial sensors to the box are those placed in the Xsens gloves. Thus, because of their positions, these sensors are not able to measure the excursion of fingertips, as shown in Figure 7. Constraining the grasp configuration or providing ergonomic handles to the loads may represent a viable solution for these cases. The highest relative error precentages are observed in the handling of the LL box: for the horizontal distance the relative error is 22.2%; for the trunk twist the relative error is 2.9%; and for the corresponding LI the relative error is 2.6%. The relative error of the trunk twist depends mainly on two factors: a starting twist offset and a shorter distance from the pallet. These considerations suggest that each subject tends to reduce, unintentionally, the risk factor by adopting a more suitable configuration in approaching the successive activity. Concerning the horizontal distance, thanks to the parameters extracted with the On-LI, it was noticed that the subjects tended to come closer to the pallet regularly during the session, as shown in Figure 8. A second look at the footage confirmed this hypothesis. This behavior is common within all subjects, and it is particularly noticeable for the boxes in positions RH and RL. For this reason, it was necessary to retake the measurement of the horizontal distance associated with the furthest stack of boxes. The reviewed measurement is reported in Table 2 as 65 cm, for row 5 and 6 of column Horizontal Distance. It is worth mentioning that differences between the observational NIOSH evaluation and the proposed On-LI architecture are not necessarily due to the poor performance of the On-LI algorithms. As a matter of fact, some of these differences may stem from the observational nature of the classic approach. For instance, differences in the subject’s starting position may have gone unnoticed with the observational method. However, the On-LI derives the NIOSH parameters relying on the digital reconstruction of the subject posture, which is assessed by the sensor data and the kinematic model. In these cases, the classic approach would have required an ergonomist to take continuous measurements, which would have implied the use of traditional tools, thus interfering with the subjects during the execution of the task. The On-LI architecture allows systematic and continuous evaluations of the Lifting Index, taking into account even slight variations in subject postures during working activities. Additionally, the On-LI architecture ensures high analysis flexibility and adaptability with respect to any possible change in the working station providing feedback and warnings with high responsiveness.

## 6. Conclusions

In this work, we presented the On-LI architecture to evaluate the ergonomic risks using a method compliant with the ISO 11228-1:2022 standard. We demonstrated the effectiveness of the proposed algorithm in identifying the NIOSH LI during lift and release phases. This innovative architecture fuses kinematic data from wearable sensors, it applies the NIOSH equations and autonomously calculates the risk factors associated with MMH activities. The algorithm relies on the standard calculation of the NIOSH Lifting Index to assess the ergonomic risk. It differs from the observational method, i.e., the ergonomist evaluates *a priori* the risk factors, because the On-LI constantly monitors worker’s postures and provides continuous appraisals of risk factors, thus accounting for any possible changes in environment variables and postures during activities.

The preliminary results of the kinematics and identification algorithm are promising, showing relative estimation errors of 2.8% in the LI with respect to the observational method, providing the prerequisites for pursuing and extending this study to a wider set of subjects also into an industrial environment. Possible improvements could target the fine estimations of the grasp positions by introducing additional sensors on fingers. Currently, the On-LI architecture has been validated with offline data because of the limitation imposed by the sensors used; however, the On-LI algorithm has been developed to work with online data.

This preliminary study revealed some possible hardware and software limitations. The former relates to the IMU technology used to reconstruct the subject posture, i.e., accelerometer, gyroscope, and magnetometer. Magnetometers could be sensitive to the environment, particularly the presence of iron frames, robots, motors, and electric power lines, which may limit the reliability of posture estimation in industrial scenarios. This will be evaluated in future work. Software limitations relate to the event detection algorithm. Here, the EDA is fine-tuned for this specific experimental protocol, to precisely detect the lift/release instants, which represent crucial parameters for assessing task-related risk factors. In future work, the EDA will include a calibration process to identify relevant thresholds, e.g., grasp/release distances and velocities, thus extending the EDA’s applicability to more complex scenarios.

In addition, future work will be conducted with the aim of (i) implementing a device able to effectively run the data analysis online, (ii) improving the estimation of the hand’s grasping position, (iii) extending the automatic detection of further NIOSH coefficients such as single- or double-limb lifting, (iv) sending online visual feedback to a user interface, (v) surveying the acceptance rate of such a system by workers.

## Figures and Tables

**Table 1:** NIOSH Lifting Index with corresponding risk magnitudes and recommendations working with healthy subjects.

Lifting Index	Risk Magnitude	Recommendation
≤1.00	Very Low	No modification required
1.01–1.50	Low	Pay attention to frequency of execution, excessively heavy loads, and/or awkward postures that are held for too long
1.51–2.00	Moderate	Redesign tasks and workplaces according to priorities to reduce the Lifting Index
2.01–3.00	High	It is highly necessary to modify the task to reduce the Lifting Index
>3.00	Very High	It is critically important to change the task immediately to reduce the Lifting Index

**Table 2:** Manually measured parameters to calculate the NIOSH ergonomic risk index. The last column reports the corresponding Lifting Index (LI). The values with an asterisk were used to overcome the issue of out-of-scale parameters, as suggested in [34].

Box	Hands Height	Vertical Travel	Horizontal Distance	Twist	Grasp	Frequency	Single Limb	Load	LI
	(cm)	(cm)	(cm)	(deg)	(G,S,P)	(Lifting/Minute)	(Yes/No)	(kg)	
FH	68	32	45	0	S	6	No	10	1.13
FL	40	88	45	0	S	6	No	10	1.33
LH	68	32	56	22	S	6	No	10	1.44
LL	40	88	56	22	S	6	No	10	1.77
RH	68	32	65 (63*)	0	S	6	No	10	1.52
RL	40	88	65 (63*)	0	S	6	No	10	1.79

**Table 3:** Automatically measured parameters to calculate the NIOSH ergonomic risk index. The last column reports the corresponding Lifting Index (LI). The values with an asterisk were used to overcome the issue of out-of-scale parameters, as suggested in [34].

Box	Hands Height	Vertical Travel	Horizontal Distance	Twist	Grasp	Frequency	Single Limb	Load	LI
	(cm)	(cm)	(cm)	(deg)	(G,S,P)	(Lifting/Minute)	(Yes/No)	(kg)	
FH	70 ± 6	34 ± 4	47 ± 8	2 ± 4	S	6	No	10	1.23 ± 0.16
FL	44 ± 5	79 ± 2	45 ± 7	3 ± 5	S	6	No	10	1.27 ± 0.16
LH	67 ± 4	38 ± 3	56 ± 7	26 ± 10	S	6	No	10	1.52 ± 0.17
LL	42 ± 4	79 ± 4	42 ± 7	24 ± 11	S	6	No	10	1.61 ± 0.26
RH	70 ± 8	39 ± 7	67 ± 5 (63*)	-2 ± 3	S	6	No	10	1.54 ± 0.09
RL	46 ± 8	75 ± 6	61 ± 6 (63*)	2 ± 3	S	6	No	10	1.69 ± 0.11

**Table 4:** Measurement errors of NIOSH parameters obtained by comparison of the On-LI algorithm and the classical method. The last column shows the resulting Lifting Index (LI) with corresponding errors.

Box	Hands Height		Vertical Travel		Horizontal Distance		Twist		LI	
	Abs. Err.	Rel. Err.	Abs. Err.	Rel. Err.	Abs. Err.	Rel. Err.	Abs. Err.	Rel. Err.	Abs. Err.	Rel. Err.
	(cm)	(%)	(cm)	(%)	(cm)	(%)	(deg)	(%)		(%)
FH	2	2.5	2	1.1	2	3.2	2	1.4	0.1	3.3
FL	4	5.0	9	5.1	0	0	3	2.2	0.06	2.0
LH	1	1.2	6	3.4	0	0	4	2.9	0.08	2.6
LL	2	2.5	9	5.1	14	22.2	2	1.4	0.15	5
RH	2	2.5	7	4.0	2	6.3	2	1.4	0.02	0.6
RL	6	7.5	13	7.4	4	3.2	2	1.4	0.1	3.3

## Data Availability

The data presented in this study are available on request from the corresponding author.

## Abbreviations

The following abbreviations are used in this manuscript:

WMSD	Work-related musculoskeletal disorder
MMH	Manual material handling
NIOSH	National Institute for Occupational Safety and Health
TACOS	Time-based assessment computerized strategy
OCRA	Occupational repetitive action
RULA	Rapid upper limb assessment
EAWS	Ergonomic assessment worksheet
On-LI	Online Lifting Index
IMU	Inertial measurement unit
EDA	Event detector algorithm
DoFs	Degree(s) of freedom
RWL	Recommended weight limit
LI	Lifting Index
MT	Motion tracker
